# Diabetes Mellitus and Its Association to the Occurrence of Medication-Related Osteonecrosis of the Jaw

**DOI:** 10.3390/dj4020017

**Published:** 2016-05-31

**Authors:** Roman K. Rahimi-Nedjat, Keyvan Sagheb, Andreas Pabst, Lukas Olk, Christian Walter

**Affiliations:** Department of Oral and Maxillofacial Surgery of the University Medical Center of the Johannes Gutenberg-University, Augustusplatz 2, 55131 Mainz, Germany; keyvan.sagheb@unimedizin-mainz.de (K.S.); andipabst@me.com (A.P.); lukasolk@gmx.de (L.O.); christian.walter@unimedizin-mainz.de (C.W.)

**Keywords:** diabetes, hyperglycemia, bisphosphonates, denosumab, MR-ONJ, BP-ONJ

## Abstract

To date there is no consensus on the role of diabetes in the development of medication-related osteonecrosis of the jaws (MR-ONJ). Therefore, this study aimed to investigate the prevalence of diabetes and pathological glucose metabolism in patients with MR-ONJ compared to the general population. All maxillofacial surgery inpatients in one year at our department were investigated regarding diagnosis, anamnesis, medication, and blood glucose readings. 1374 records were analyzed. 35 patients with MR-ONJ were identified. Diabetics accounted for 14.3%. No significant difference in the prevalence of known diabetes was found, except for pathological glucose metabolism in patients with MR-ONJ (*p* < 0.001). Diabetes does not necessarily promote the onset of MR-ONJ. Therefore, diabetes should not be considered as a standalone risk factor. On the contrary, hyperglycemia as a possible indicator for poorly managed or yet undetected diabetes is associated with MR-ONJ.

## 1. Introduction

Since its first description slightly more than a decade ago bisphosphonate-associated osteonecrosis of the jaws (BP-ONJ) has become one of the main issues in current oral and maxillofacial surgery [1,2]. Depending on the different indications, bisphosphonate potency, type of administration, duration of exposure, and co-medication the incidence of BP-ONJ differs substantially from 0.1% to 20% for subgroups with further risk factors. In addition to this a RANKL-inhibitor (Receptor Activator of NF-κB Ligand) has been added to the market and seems to lead to even higher incidences of medication-related osteonecrosis of the jaws (MR-ONJ) compared to bisphosphonates. The term MR-ONJ nowadays is linked to necrosis due to medication with bisphosphonates, denosumab, and also other drugs (e.g., antiangiogenic drugs) [1,2,3,4,5,6,7,8].

MR-ONJ is most probably caused by multiple factors, including reduced bone remodeling, and negative impacts of medication on soft tissue cells and antiangiogenic properties [9,10,11].

Usually MR-ONJ is triggered by inflammatory processes such as periodontal diseases, poor oral hygiene, or surgical procedures with subsequent inflammation due to the wound healing process [12]. Furthermore, systemic factors such as age, co-medications (e.g., chemo- or cortison-therapy), nutritional conditions, and alcohol and tobacco intake seem to influence the course of the disease [5,7,10].

Diabetes mellitus (DM) alters the immune response and lowers the resistance to infections both for cutaneous and oral wounds [13,14]. The antiangiogenic and negative effect on the soft tissues of an antiresorptive medication in combination with diabetic micro- and macrovascular changes could theoretically lead to impaired wound healing and therefore promote the onset of MR-ONJ [15,16,17].

However, to date there is still no consensus on the influence of DM on MR-ONJ. The aim of this study was therefore to investigate the relationship between diabetes, hyperglycemia, and MR-ONJ.

## 2. Material and Methods

A cross-sectional study was conducted in which electronic health records of all patients who received inpatient treatment during one year (1 January 2013 to 31 December 2013) at the Department of Oral and Maxillofacial Surgery of the Medical Center of the Johannes Gutenberg-University of Mainz were investigated.

Records were screened and details regarding epidemiologic data, anamnesis, and diagnosis were noted. Special attention was given to diabetes anamnesis as well as medication histories spotlighting bisphosphonates and denosumab.

MR-ONJ was noted for patients presenting with osteonecrosis, sequester or bone infection with a previous or ongoing bisphosphonate or denosumab treatment with no history of head and neck radiation. All patients underwent clinical, radiological, and histopathological examination.

All maximum blood glucose readings were analyzed and values above 200 mg/dL at any time were considered pathological [15].

The whole collective of maxillofacial surgery patients treated in the same time span was chosen as a control-group. Therefore, inpatients without MR-ONJ receiving treatment due to any other diagnosis were included in the control-group.

The statistical analysis was performed using SPSS 23.0 (IBM; Chicago, IL, USA) using the Χ^2^-test for binary values and *t-*test for mean values. *p*-values of less than 0.05 were considered statistically significant.

## 3. Results

Altogether 1374 patients were treated in the year 2013 (average age 47.98 ± 23.74 (standard deviation), 60.0% male (46.68 years ± 23.11) and 40.0% female (49.93 years ± 24.55)). Of these 35 patients 2.54% had MR-ONJ (average age 64.94 years ± 8.37, 34.3% male (68.75 years ± 8.14) and 65.7% female (62.96 years ± 7.95)). There was a statistically significant difference (*p* > 0.001) regarding the age of the patients with and without MR-ONJ (Table 1).

Of all patients 8.7% (*n* = 120) had a history of diabetes mellitus type I or II (average age 69.07 years ± 13.21, 67.5% male (67.17 years ± 13.02) and 32.5% female (73.0 years ± 12.89)). 74.1% (*n* = 89) of these received medication while the rest (25.8%, *n* = 31) were on dietary treatment. The most prescribed medication was insulin (42.7% of the patients, *n* = 38), followed by metformin (34.8%, *n* = 31) and sulfonylurea (15.7%, *n* = 14). The most frequently recorded diabetic comorbidity was nephropathy in 30% of the patients (*n* = 36), followed by neuropathy (14.2%, *n* = 17).

Of the 35 patients with MR-ONJ 5 14.3% were diabetics 14.3%. All of them received medicamentous treatment mostly with metformin (40.0%, *n* = 2) and insulin (40.0%, *n* = 2). Nephropathy was seen in three out of five cases and retinopathy in one case.

No statistical difference could be detected for the prevalence of diabetes in patients with MR-ONJ compared to the control-group (*p* = 0.223).

The average of all maximum blood glucose readings was 105 mg/dL; 11.4% (*n* = 142) showed values above 200 mg/dL out of which 56.3% (*n* = 80) were known diabetics. Among MR-ONJ-patients the average maximum value was 113 mg/dL. Of these values, 28.5% (*n* = 10) were above 200 mg/dL. A history of diabetes was found in only four out of 10 patients. We found a strongly significant relationship between the prevalence of pathological values and MR-ONJ (*p* < 0.001).

## 4. Discussion

Several studies have been published in the last few years investigating the relationship between diabetes and osteonecrosis of the jaws due to antiresorptive medication such as bisphosphonates or denosumab. As there is still no consensus, our study aimed to investigate the prevalence of diabetes and pathological glucose metabolism in inpatients treated for MR-ONJ in our department during one year. The whole collective of maxillofacial surgery patients treated in the same year for any other diagnosis served as a control group.

In 2007 Khamaisi *et al.* described a significant difference in the prevalence of diabetes in patients with BP-ONJ in comparison to patients with bisphosphonates-treatment without osteonecrosis. Of 31 patients 58% had a history of diabetes or impaired fasting glucose [18]. Another study describes a proportion of 86.6% of diabetics among 25 patients with osteonecrosis. However, there seems to be a mistake in the description of the study since the authors state that an incomprehensible 168 diabetics are among these 25 patients, which obviously is impossible, therefore this study should be handled with care [19].

A recently published review states that most studies investigating the relationship between DM and MR-ONJ report a positive association [7]. Therefore, we conducted a search for all clinical studies contributing to this topic and found 11 articles (Table 2). Out of 12 studies, including the present study, four showed a significant relationship between diabetes and MR-ONJ, among them the paper with the inconclusive description of the results [18,19,20,21]. Another four studies did not calculate *p*-values [22,23,24,25] and the remaining four studies did not show a statistical significance [26,27,28]. One of these is the investigation by Wilkinson *et al.* and represents the biggest collective with 16,073 included patients [29]. After reviewing the literature we cannot assume a standalone impact for diabetes on MR-ONJ.

Despite this, most studies do refer to the higher prevalence of diabetes in patients with MR-ONJ compared to the normal population. Based on this, they conclude there is an important role for diabetes in the course of the disease [22]. Our study showed a considerable difference in the number of diabetics as well with an almost twofold higher prevalence in patients with MR-ONJ (14.3% *vs.* 8.7%). However it is important to consider the age difference between the collectives. The most important diagnoses leading to treatment with antiresorptive medication are either metastatic malignancies or osteoporosis [1,2,3,10]. These diseases are, however, not frequently found in young patients, but rather come along with higher age at the time of diagnosis. Diabetes as one of the main epidemics of the 21st century is a main issue for patients above 45 years in developing countries and above 64 years in industrial countries [30]. Consequentially, the high prevalence of diabetes in this targeted group can be attributed to the higher age of the patients. Moreover, it has to be taken into account that diabetes itself has been described as a risk factor for osteoporosis [31]. In the present study, the average age and the proportion of patients with diabetes were higher in patients with MR-ONJ compared to the control group, also showing this relationship. Furthermore, the mean age of all reviewed studies for MR-ONJ patients is 67.91, which also confirms the age difference between the reviewed group and the control population.

Currently, many studies can be found that report no difference in the treatment outcome between non-diabetics and patients with well-controlled diabetes [32,33]. A blood glucose value above 200 mg/dL at any time, however, can be defined as hyperglycemic and can be an indicator of poorly managed diabetes [15]. Hyperglycemia might impact bone metabolism in several ways, as it is known to perpetuate inflammatory alterations and promote lactate accumulation [34,35]. Our findings support this theory, as we calculated a statistically significant relationship between hyperglycemia and MR-ONJ. Interestingly, a large proportion of patients with high pathological blood glucose values did not have a history of diabetes or had never received the therapeutic recommendations described in literature before [15]. Therefore, in our opinion, it is important to control pathological glucose metabolism in order to potentially have an influence on the development and/or course of the disease.

## 5. Conclusions

Our study did not show a significant difference in the prevalence of diabetes between patients with MR-ONJ compared to a general collective of oral and maxillofacial surgery patients. Based on our results and those we found in the review of the literature, we have to assume that a diagnosis of diabetes mellitus is not associated with the onset of an osteonecrosis due to antiresorptive medication. On the contrary, we agree with the current literature on diabetes management and believe that hyperglycemia, as a possible indicator for poorly managed diabetes, has a significant impact on the course of MR-ONJ.

## Figures and Tables

**Table 1:** Overview of patients with medication-related osteonecrosis of the jaws (MR-ONJ) and the control-group.

Values	Control Group	MR-ONJ	*p*-Value
*n*	1339	35	
Age mean	47.53	64.94	*p* < 0.001
Age min	0	45	
Age Max	99	88	
Age standard deviation	23.85	8.37	
Known diabetes (%)	115 (8.6)	5 (14.3)	*p* = 0.223
Diabetes therapy			
Dietary (%)	31 (27.0)	0	
Medicinal (%)	84 (73.0)	5 (100.00)	
Nephropathy (%)	33 (28.7)	3 (60.0)	
Increased Creatinine level (%)	14 (12.1)	0	
Neuropathy	17 (14.8)	0	
Retinopathy	5 (4.3)	1 (20.0)	
Diabetic foot ulcer	4 (3.5)	0	
Gangrene	1 (0.9)	0	
Metformin	29 (25.2)	2 (40.0)	
Glinide	0	0	
Acarbose	0	0	
Sulfonylurea	14 (12.1)	1 (20.0)	
Glitazone	0	0	
Gliptin	4 (3.5)	0	
Insulin	38 (33.0)	2 (40.0)	
Average maximum blood glucose level (mg/dL)	105	113	
Number of patients with glucose values above 200 mg/dL (%)	142 (11.4)	10 (28.5)	*p* < 0.001
Number of diabetics (%)	80 (56.3)	4 (40)	

**Table 2:** Studies published concerning the relationship between diabetes and MR-ONJ. Where BP-ONJ refers to bisphosphonate-associated osteonecrosis of the jaws.

Study	Country	No. of Patients	Prevalence of Diabetes (%)	*p*-Value	Average Age	Comment
Vidal-Real, 2015 [19]	Spain	194	86.6	0.048	68.91	only patients treated with zoledronic acid; comparison BP-ONJ and BP-Treatment
Khamaisi, 2007 [18]	Israel	31	58	0.001	64.8	comparison BP-ONJ and BP-Treatment
Bocanegra-Perez, 2012 [22]	Spain	44	35	not calculated	64.2	
Fede, 2013 [26]	Italy	87	9.2	not significant	70.7	osteoporotic non-cancer patients
Anavi-Lev, 2013 [20]	Israel	52	41 *	0.02	74.5	comparison between iv and po BP-treatment; diabetes prevalence higher in po-group
Diniz-Freitas, 2012 [23]	Spain	20	20	not calculated	71.2	
Lazarovici, 2009 [24]	Israel	101	16	not calculated	63.5	
Watters, 2013 [21]	USA	154	24	0.05	64	only BP-ONJ-patients included, comparison between progressive disease and remission
Manfredi, 2011 [25]	Italy	25	16	not calculated	70.4	only patients with BP-treatment due to osteoporosis
Wilkinson, 2007 [29]	USA	16073	6.40	not significant	n/a	
Molcho, 2013 [27]	Israel	46	37	not significant	66	
present study, 2016	Germany	35	14.30	not significant	68.8	
			30.29		67.91	

* value recalculated.
